# INFLA score: a novel inflammatory marker for assessing cardiometabolic disease risk in obese individuals

**DOI:** 10.1186/s13098-024-01396-8

**Published:** 2024-07-09

**Authors:** Shuke Liu, Yan Gu

**Affiliations:** Department of Cardiovascular Medicine, Affiliated Hospital of Xuzhou Medical University, No. 99 Huaihai Road, Xuzhou, 221000 Jiangsu China

**Keywords:** INFLA-score, Low-grade inflammation, Obesity, Cardiometabolic diseases, Inflammatory markers, UK Biobank

## Abstract

**Background:**

The low-grade inflammation score (INFLA-score) is a composite index that assesses chronic inflammatory status using multiple inflammatory markers. However, its correlation with cardiometabolic diseases (CMDs) in obese populations remains unclear.

**Methods:**

We conducted a prospective cohort study involving 79,160 participants with obesity (BMI ≥ 30 kg/m^2^) from the UK Biobank. The INFLA-score was calculated based on high-sensitivity C-reactive protein, leukocyte count, platelet count and granulocyte/lymphocyte ratio. We employed Kaplan–Meier survival curves, multivariable Cox regression, restricted cubic splines and accelerated time-to-failure models to analyse the association between the INFLA-score and CMDs risk, including coronary heart disease (CAD), stroke and type 2 diabetes mellitus (T2DM).

**Results:**

Over a median follow-up of 161.41 months, we recorded 14,903 CMDs events, comprising 7184 CAD cases, 1914 strokes and 7924 T2DM cases. Cox regression analysis revealed that each unit increase in the INFLA-score corresponded to a 1.5%, 1.1%, 1.2% and 2.4% increase CMDs risk (HR: 1.015, 95% CI 1.013–1.018), CAD risk (HR: 1.011, 95% CI 1.007–1.015), stroke risk (HR: 1.012, 95% CI 1.004–1.020) and T2DM risk (HR: 1.024, 95% CI 1.020–1.028), respectively. Restricted cubic spline analysis indicated a non-linear relationship between cumulative INFLA-score and CMDs risk (P = 0.044). Subgroup analysis revealed interactions between sex, age, history of lipid-lowering drug use, and INFLA-score regarding CMDs risk. Sensitivity analysis corroborated the main findings.

**Conclusion:**

Our findings strongly support the close association between INFLA-score and CMDs risk, particularly notable in women, those aged < 55, and individuals with a history of lipid-lowering drug use. These findings offer new insights into the role of inflammation in obesity-related CMDs, suggesting potential applications for prevention and identification of high-risk populations.

**Supplementary Information:**

The online version contains supplementary material available at 10.1186/s13098-024-01396-8.

## Introduction

Obesity, defined as a body mass index (BMI) equal to or exceeding 30 kg/m^2^, is characterised by excessive fat accumulation or abnormal distribution [1]. Globally, obesity rates have been steadily rising over the past four decades, with particular concern for the significant increase among young individuals. Presently, over 100 million children and 600 million adults are affected, and projections suggest that by 2025, global obesity rates will soar to 18% in males and 21% in females [2, 3]. This trend poses a substantial threat to public human health and safety. Studies indicate that for individuals with a BMI exceeding 25 kg/m^2^, every 5 kg/m^2^ increase raises the risk of all-cause mortality by 30% [4]. Additionally, obesity is closely linked to the onset of cardiometabolic diseases (CMDs) such as coronary artery disease (CAD), type 2 diabetes mellitus (T2DM) and stroke [2].

Chronic low-grade inflammation accompanies obesity, where pro-inflammatory cytokines disrupt insulin signalling in peripheral tissues via autocrine and paracrine pathways, leading to metabolic disorders and insulin resistance (IR) [5, 6]. These factors significantly contribute to the development of CMDs [7]. Previous studies have identified several indicators, including vitamin D, high-sensitivity C-reactive protein (hs-CRP) and leukocyte count, to assess inflammation levels, showing significant associations with metabolic diseases (Mets) and CMDs [8–10]. However, these indicators operate independently, with potential anti-inflammatory or pro-inflammatory functions in different biological processes, lacking a multidimensional assessment method to consider their combined effects [11].

The low-grade inflammation index (INFLA-score) is a composite index based on hs-CRP, platelet count, leukocyte count and granulocyte-to-lymphocyte ratio (GrL), encompassing different components of the inflammatory response process [12]. Compared to individual indicators, the INFLA-score offers a more comprehensive evaluation of combined inflammatory markers, providing an accurate measure of the body's inflammatory response. Several studies have demonstrated the utility of the INFLA-score in various populations. For instance, Crotti et al. reported a 1% increase in overall mortality risk for each unit increase in the INFLA-score (HR 1.01, 95% confidence intervals (CI): 1.01–1.52) [13], with higher INFLA-scores elevating mortality risk among obese elderly individuals [13]. Moreover, following anti-inflammatory treatment, reduction in the INFLA-score significantly downregulates 8-oxoGuo [14], associated with key risk factors such as diabetes, hypertension and cardiovascular diseases (CVDs) [15, 16].

Despite these advancements, research on the relationship between INFLA-score and CMDs in individuals with obesity remains largely unexplored. Therefore, we aim to conduct a large-scale prospective study to investigate the association between INFLA-score and CMDs, including CAD, stroke and T2DM, in individuals with obesity, thereby addressing the existing research gap and providing crucial insights into the role of INFLA-score in the pathogenesis of CMDs.

## Method

### Data source and study design

The UK Biobank, a large-scale prospective cohort study conducted across the United Kingdom from 2006 to 2010, served as the data source. Detailed methodology for the study has been extensively described in prior publications [17].

Baseline data, including demographic and clinical characteristics, lifestyle factors and health information, were collected through physical assessments, interviews and laboratory tests. Ethical approval was obtained from the North West Multi-Centre Research Ethics Committee (REC No. 11/NW/0382), and all participants provided informed consent. Further information about the UK Biobank can be accessed on the official website at www.ukbiobank.ac.uk.

Initially, 89,011 obese individuals were identified based on a BMI ≥ 30 kg/m^2^. Participants with complete INFLA-score data and no prior CMDs (including CAD, stroke and T2DM) were included. Exclusions were made for individuals with cancer (n = 8052) and pregnancy (n = 25) at baseline, as well as those with less than 2 years of follow-up (n = 1774) to mitigate the impact of reverse causality. Ultimately, 79,160 participants were included in the analysis. The study adhered strictly to the ethical guidelines of the Declaration of Helsinki for medical research involving human subjects.

### Assay of inflammatory biomarkers

Blood samples collected during the initial recruitment phase were analysed at the UK Biobank Central Laboratory within 24 h. Complete blood counts were determined using an LH750 haematology analyzer (Coulter, Beckman Coulter, Brea, CA, USA), while high-sensitivity immunoturbidimetric analysis on a Beckman Coulter AU5800 analyzer (Beckman Coulter (UK) Ltd) quantified hs-CRP levels.

### Calculation of the INFLA-score

The INFLA-score comprised four components: hs-CRP, leukocyte count, platelet count and GrL. Granulocyte count includes the sum of neutrophils, eosinophils and basophils. Biomarkers were categorised into scoring levels based on their decile distribution: scores of + 1 to + 4 for values in the upper deciles (7th to 10th); 0 for measurements in the middle deciles (5th and 6th); and scores of − 4 to − 1 for values in the lower deciles (1st to 4th). The total INFLA-score, ranging from -16 to + 16, represents an individual’s level of low-grade inflammation [18, 19], with higher scores indicating a more pronounced inflammatory status.

### Assessment of other variables

Baseline surveys provided self-reported data on various parameters, including age, sex, ethnic background, blood pressure, lipid profiles, physical activity, Townsend Deprivation Index (TDI), existing chronic conditions, medication use and smoking and drinking habits. The TDI, a measure of socioeconomic status, incorporates variables such as employment status, car and home ownership and household overcrowding, with higher values indicating lower socioeconomic status. Dietary risk factors were assessed using a cumulative dietary risk score based on nine food items (processed meat, red meat, fish consumption, milk, spreads, cereals, salt intake, water intake and fruit and vegetable consumption), categorised according to compliance with UK and European dietary guidelines [20]. Participants received one point for each instance of unhealthy consumption, culminating in a dietary score ranging from 0 (healthiest diet) to 9 (least healthy diet). Physical activity levels were quantified in total metabolic equivalent (MET) minutes per week using a modified version of the International Physical Activity Questionnaire [21]. Additionally, BMI, blood pressure and lipid profiles were measured following established protocols [22]. Hypertension was identified based on hospitalisation diagnosis, antihypertensive medication uses and self-reported data.

### Study outcomes

The primary endpoint was the incidence of CMDs, encompassing CAD, T2DM onset and stroke collectively. The secondary endpoint included the onset of CAD, T2DM and stroke. Diagnostic algorithms were developed to identify these conditions, utilising information from death registries, primary care, hospitalisation records and self-reported data. CMDs diagnoses were based on the International Classification of Diseases, Tenth Revision (ICD-10) codes. CAD was identified by codes I20-25, T2DM by code E11, hypertension by code I10 and stroke by codes I60-64. Data updates were last made on 21 November 2022, for CAD, 29 November 2022 for stroke, and 1 April 2023 for T2DM. Each participant's observation period extended from their cohort enrolment date until the first occurrence of CMDs, death, or censoring, whichever came first.

### Statistical analysis

Missing categorical variables were treated as missing indicators, while continuous variables were estimated as means. Data normality was assessed using the Kolmogorov–Smirnov test, which indicated that variables did not follow a normal distribution. Categorical variables were presented as frequencies and percentages, while continuous variables were expressed as medians and interquartile ranges (IQRs). Quartiles of INFLA-scores were determined, and group differences were evaluated using the Chi-squared test for categorical variables and the Kruskal–Wallis test for continuous variables. Incidence rates of CMDs in quartile groups were expressed as units per 1000 person-years, with cumulative incidence presented as a percentage, and p-values for the trend in between-group changes were calculated using Poisson regression.

Kaplan–Meier survival curves were employed to estimate CMD incidence in INFLA quartile groups, with differences between groups assessed using log-rank tests. HRs and 95% CIs for the association between INFLA-scores and CMD risk were calculated using the Cox proportional hazards model. The proportional hazards assumption was evaluated using the Schoenfeld Residuals test, with no violations detected. Three Cox regression models were constructed: Model 1 was a crude model with no adjustment for variables; Model 2 was adjusted for age, sex and ethnicity; and Model 3 was further adjusted for hypertension, TDI, BMI, DBP, SBP, physical activity, TC, HbA1c, HDL, LDL, diet score, TG, smoking and alcohol status, antihypertension and lipid levels. In Cox regression models, INFLA-scores were evaluated as both continuous and categorical variables in relation to CMDs risk, with trend analysis conducted between categorical groups. In addition, Z-score normalisation (mean = 0, standard deviation (SD) = 1) quantified the change in CMDs risk per SD unit increase in the INFLA-score, as well as its constituent inflammatory indicators. Restricted cubic spline (RCS) with three nodes (10th, 50th and 90th percentiles) were used to visually assess the dose–response relationship between INLFA-scores and CMDs risk, with non-linear associations identified using the log-likelihood ratio test. If non-linear associations were observed, a two-part linear regression model was further used to determine the inflexion point at which the relationship changed significantly. Detailed analyses are presented in Appendix 1.

Subgroup analyses were conducted based on clinical characteristics: age (< 55 and ≥ 55 years), sex (men and women), ethnicity (Caucasian and non-Caucasian), BMI (30–34.99, 35–39.99, and ≥ 40 kg/m^2^), hypertension, and lipid-lowering medications (yes and no), with p-values calculated for between-group interactions using the likelihood ratio test.

Additionally, the accelerated time to failure (AFT) model was employed to assess the potential effects of different INFLA-score groups on the time to CMDs onset. The model assumes that covariates accelerate or delay the time trajectory of events and does not rely on proportional risk assumptions, highlighting its potential application in diverse clinical studies [23, 24]. In a multivariate AFT model, the effect of increasing quartiles of INFLA-scores on the time to onset of CMDs was assessed using the first INFLA-score quartile (Q1) as the reference group. The difference in median time to CMDs onset between the two groups was measured in months and calculated by subtracting the comparison group from Q1. Negative values indicated delayed onset of CMDs while positive values indicated earlier onset. In this study, we selected a flexible ‘Weibull distribution’ to accommodate the right-skewed shape of CMDs onset time (Figures S1 and S2).

In sensitivity analyses, we verified the stability of the results using two strategies. First, missing values were estimated using multiple imputations. Missing variables were estimated using a five-repeat prediction-means matching algorithm and a Markov chain Monte Carlo method, and pooled analyses were performed with a Cox regression model. Second, the main analysis was reanalysed for participants with less than 2 years of follow-up. Statistical analyses were conducted using R software (version 4.2.0), with statistical significance set at two-sided P < 0.05.

## Results

### Population characteristics

A total of 79,160 participants with obesity were included, with a mean age of 56 years (49, 62 kg/m^2^) and a mean BMI of 32.59 kg/m^2^ (IQR, 31.09–35.12 kg/m^2^), of whom 49.39% were men. Over 94% of the participants were Caucasian. (Table S1). Baseline characteristics stratified by quartiles of INFLA-score are presented in Table 1. In the highest quartile of INFLA-score (Q4), women, Caucasian ethnicity, current smokers, individuals with hypertension, users of antihypertensive medication, individuals with diabetes, BMI, DBP, HbA1c, hs-CRP, leukocyte count, platelet count and GrL levels were significantly higher than other groups (all P < 0.001). Conversely, current drinkers, users of lipid-lowering medication and physical activity were lower in the Q4 group compared to other quartiles (all P < 0.001).

**Table 1:** Baseline characteristics of INFLA-score quartile levels

INFLA quartile	Q1 (− 16 to − 5) N = 19,651	Q2 (− 4 to − 1) N = 18,065	Q3 (0 to 3) N = 18,101	Q4 (4 to 16) N = 23,343	P-value
Age	57 (50, 62)	57 (50, 62)	57 (50, 62)	56(48, 62)	< 0.001
Men (%)	11,401 (58.02%)	8933 (49.45%)	7902 (43.66%)	7693 (32.96%)	< 0.001
Caucasian (%)	17,716 (90.80%)	16,974 (94.45%)	17,214 (95.48%)	22,304 (95.98%)	< 0.001
Current smoker (%)	1252 (6.41%)	1393 (7.75%)	1831 (10.18%)	2935 (12.65%)	< 0.001
Current drinker (%)	17,997 (91.93%)	16,578 (91.97%)	16,532 (91.56%)	20,821 (89.47%)	< 0.001
Hypertension (%)	6416 (32.65%)	6523 (36.11%)	6686 (36.94%)	9306 (39.87%)	< 0.001
Antihypertensive (%)	4300 (21.93%)	4535 (25.13%)	4725 (26.13%)	6821 (29.25%)	< 0.001
Lowering lipids (%)	3059 (15.60%)	2885 (15.99%)	2712 (15.00%)	3190 (13.68%)	< 0.001
TDI	− 1.95 (− 3.53, 0.83)	− 1.93 (− 3.5, 0.85)	− 1.82 (− 3.46, 1.06)	− 1.52 (− 3.3,1.47)	< 0.001
BMI (Kg/m^2^)	31.97 (30.84, 33.89)	32.34 (31.00, 34.56)	32.66 (31.13, 35.17)	33.53 (31.52, 36.8)	< 0.001
DBP (mmHg)	86 (79.5, 92)	86 (80, 92.5)	86.5 (80.5, 93)	86.71 (80.5, 93)	< 0.001
SBP (mmHg)	139.5 (129.00, 151.5)	140.5 (129.50, 152.5)	140.50 (130, 153)	140.5 (129.5, 153)	< 0.001
Physical activity (MET-min/week)	2364.68 (973.00, 2750.5)	2364.68 (918, 2555)	2364.68 (866, 2364.68)	2346 (753, 2364.68)	< 0.001
TC (mmol/L)	5.71 (4.98, 6.47)	5.75 (5.05, 6.50)	5.81 (5.09, 6.57)	5.78 (5.08, 6.53)	< 0.001
HDL (mmol/L)	1.3 (1.1, 1.5)	1.3 (1.1, 1.5)	1.3 (1.1, 1.5)	1.3 (1.1, 1.5)	0.002
LDL (mmol/L)	3.66 (3.09, 4.23)	3.67 (3.13, 4.24)	3.71 (3.16, 4.29)	3.70 (3.15, 4.27)	< 0.001
TG (mmol/L)	1.74 (1.23, 2.48)	1.85 (1.32, 2.58)	1.90 (1.37, 2.64)	1.89 (1.38, 2.61)	< 0.001
HBA1C	35.50 (33.00, 37.6)	35.90 (33.3, 38.1)	36.24 (33.7, 38.4)	36.30 (34.2, 39)	< 0.001
Glucose	5.03 (4.69, 5.3)	5.03 (4.68, 5.3)	5.02 (4.67, 5.3)	5.02 (4.65, 5.33)	0.501
Diet score	5 (4, 6)	5 (4, 6)	5.00 (4, 6)	5.00 (4, 6)	< 0.001
Inflammation-related biomarkers					
C-reactive protein (mg/L)	1.25 (0.78, 1.99)	2.03 (1.28, 3.23)	2.83 (1.78, 4.53)	5.20 (3.18, 8.85)	< 0.001
Leucocyte count (10^^9^ cells/L)	5.70 (5.04, 6.41)	6.62 (5.90, 7.40)	7.30 (6.55, 8.19)	8.58 (7.60, 9.70)	< 0.001
Platelet count (10^^9^ cells/L)	214.10 (187.40, 242.4)	241.9 (213.00, 272.2)	261.00 (231.80, 294)	296 (260.70, 333.9)	< 0.001
Lymphocyte count (10^^9^ cells/L)	1.92 (1.60, 2.3)	2 (1.61, 2.4)	2.02 (1.67, 2.5)	2.10 (1.70, 2.52)	< 0.001
Neutrophil count (10^^9^ cells/L)	3.14 (2.70, 3.61)	3.9 (3.43, 4.4)	4.50 (3.99, 5.07)	5.58 (4.88, 6.44)	< 0.001
Eosinophil count (10^^9^ cells/L)	0.12 (0.1, 0.2)	0.15 (0.1, 0.21)	0.17 (0.1, 0.24)	0.20 (0.1, 0.28)	< 0.001
Basophil count (10^^9^ cells/L)	0.02 (0, 0.03)	0.02 (0, 0.04)	0.03 (0, 0.05)	0.03 (0, 0.07)	< 0.001
GrL	1.7 (1.4, 2.06)	2.07 (1.71, 2.5)	2.32 (1.92, 2.84)	2.82 (2.3, 3.48)	< 0.001
INFLA-score Components					
C-reactive protein score	− 2 (− 4, − 1)	0 (− 2, 1)	0 (− 1, 2)	2 (0,4)	< 0.001
Leucocyte count score	− 3 (− 4, − 1)	− 1 (− 2, 0)	0 (− 1, 2)	2. (1, 4)	< 0.001
Platelet count score	− 2. (− 3, 0)	0 (− 2, 1)	0 (− 1, 2)	2 (0, 3)	< 0.001
GrL score	− 2 (− 4, 0)	0 (− 2, 1)	0.00 (− 1, 2.)	2. (0, 3)	< 0.001

The INFLA-score values are all integers and therefore cannot be fully equalized when grouping at the quartile level

INFLA: the aggregated inflammation score; TDI: Townsend Deprivation Index; BMI: body mass index; SBP: systolic blood pressure; DBP: diastolic blood pressure; TC: total cholesterol; TG: triglyceride; LDL-C: low-density lipoprotein cholesterol; HDL-C: high-density lipoprotein cholesterol; MET: metabolic equivalent task; GrL: granulocyte to lymphocyte ratio. Granulocytes are the sum of the cell counts of neutrophils, eosinophils, and basophils

### INFLA-score and CMDs events

**Fig. 1:**
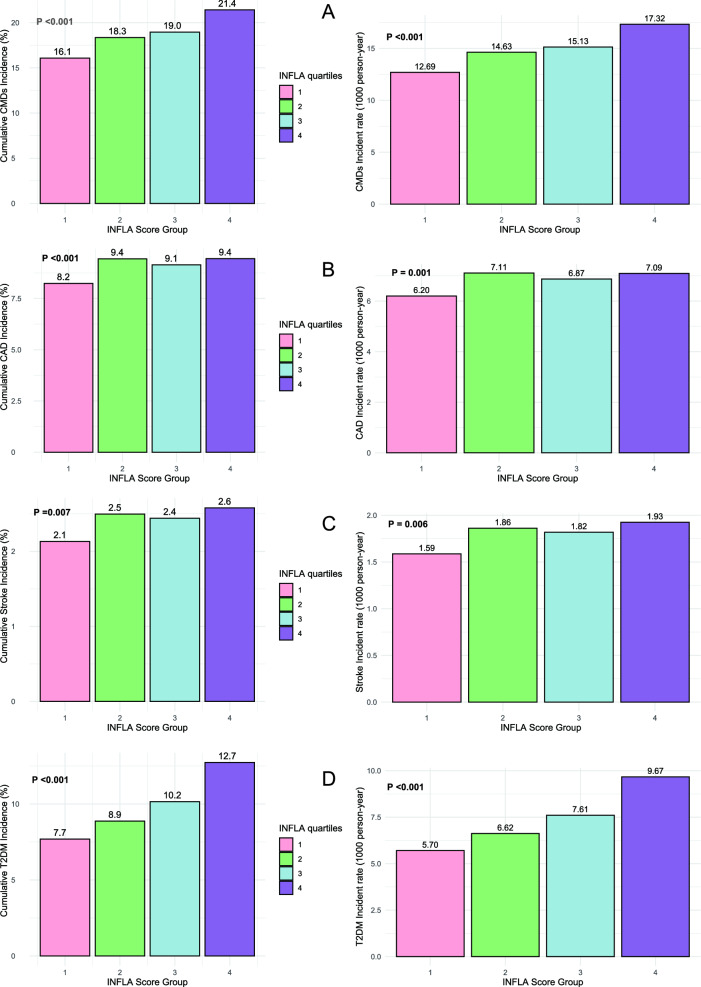
The incidence of CMDs in INFLA-score quartile groups. **A** Cardiometabolic diseases (CMDs). **B** Coronary heart disease (CAD). **C** Stroke. **D** Type 2 diabetes mellitus (T2DM)

**Fig. 2:**
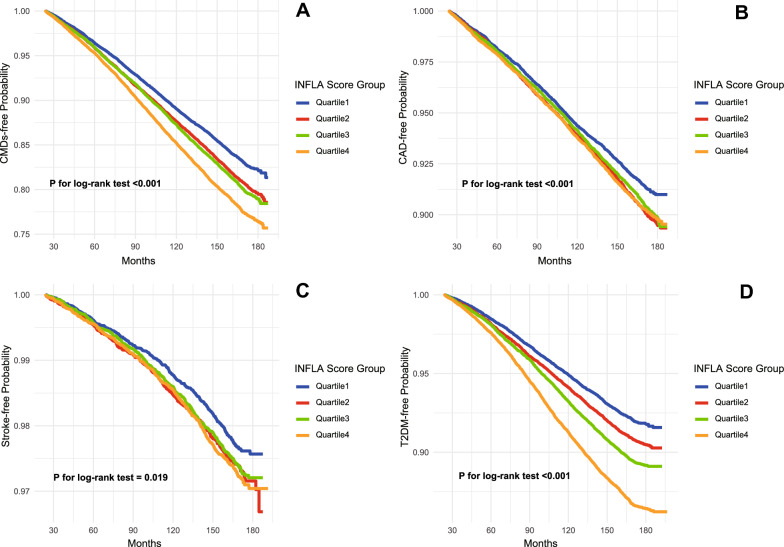
Kaplan–Meier survival curve for CMDs across the INFLA-score quartile groups. **A** Cardiometabolic diseases (CMDs). **B** Coronary heart disease (CAD). **C** Stroke. **D** Type 2 diabetes mellitus (T2DM). Hazard ratios were adjusted for the same variables included in model 3

Over a median follow-up of 161.41 months (IQR, 24.03–187.33), a total of 14,903 CMDs events were observed, including 7184 cases of CAD, 1914 strokes and 7924 cases of T2DM. Across quartiles of INFLA-score, the total incidence rates of CMDs were 12.69, 14.63, 15.13 and 17.32 per 1000 person-years in Q1, Q2, Q3 and Q4 groups, respectively (P < 0.001, Fig. 1). Similarly, the incidence rates of CAD were 6.2, 7.11, 6.87 and 7.09 (P = 0.001); those of stroke were 1.59, 1.86, 1.82 and 1.93 (P = 0.006, Fig. 1); and those of T2DM were 5.7, 6.62, 7.61 and 9.67 (P < 0.001, Fig. 1). The Kaplan–Meier curves demonstrated a progressive increase in the incidence of CMDs from Q1 to Q4 (all P for log-rank test < 0.05, Fig. 2).

### Association between the INFLA-score and CMDs risk

HRs (95% CIs) for the association between quartiles of INFLA-score and CMDs risk are presented in Table 2. In unadjusted Cox regression analyses, higher INFLA-scores were associated with increased risks of CMDs, CAD, stroke and T2DM. These associations remained significant after adjusting for age, sex and ethnicity (Model 2). In Model 3, compared to the lowest quartile (Q1) of INFLA-score, the CMDs risks for the incremental quartiles of INFLA score were 1.09 (1.04–1.15), 1.13 (1.08–1.19), and 1.25 (1.19–1.31), respectively. Moreover, a significant upward trend was observed in the quartiles of INFLA-score, indicating a significant increase in the risk of total CMDs with increasing quartiles of INFLA-score (all P trend < 0.001). Similar results were observed in the fully adjusted models of CAD, stroke and T2DM. In Model 3 adjusted for CAD, stroke and T2DM, the HRs for the highest quartiles of INFLA-score were 1.18 (1.11–1.27), 1.24 (1.09–1.42) and 1.38 (1.29–1.48), respectively, compared to the lowest quartiles. The trend between quartiles of INFLA-score and the risk of CAD, stroke and T2DM was significant (P for trend < 0.01). Each unit increase in INFLA-score was associated with a 1.5% increase in total CMDs risk (HR: 1.015, 95% CI 1.013–1.018), a 1.1% increase in CAD risk (HR: 1.011, 95% CI 1.007–1.015), a 1.2% increase in stroke risk (HR: 1.012, 95% CI 1.004–1.020) and a 2.4% increase in T2DM risk (HR: 1.024, 95% CI 1.020–1.028). Additionally, INFLA score as well as the constituent inflammatory indicators were Z-value normalised, in order to further compare their association with CMDs risk. The results showed that with each 1-SD unit increase in INFLA score, hs-CRP, leukocyte count, and GrL, the risk of CMDs increased by 9.9%, 7.3%, 9.6%, and 2.7%, respectively (all P < 0.01; Table S2). However, each SD unit increase in platelet count was not significantly associated with the risk of CMDs (P = 0.945; Table S2).

**Table 2:** Multivariate Cox regression of INFLA-score and CMDs risk

	Model 1		Model 2		Model 3	
	HR (95% CI)	P value	HR (95% CI)	P value	HR (95% CI)	P value
**INFLA score**			**CMDs**			
Quartile 1	Ref		Ref		Ref	
Quartile 2	1.16 (1.10–1.21)	< 0.001	1.21 (1.15–1.27)	< 0.001	1.09 (1.04–1.15)	< 0.001
Quartile 3	1.20 (1.14–1.26)	< 0.001	1.31 (1.25–1.37)	< 0.001	1.13 (1.08–1.19)	< 0.001
Quartile 4	1.38 (1.32–1.44)	< 0.001	1.65 (1.58–1.73)	< 0.001	1.25 (1.19–1.31)	< 0.001
P for trend	< 0.001		< 0.001		< 0.001	
Per unit increase	1.020 (1.018–1.023)	< 0.001	1.034 (1.031–1.037)	< 0.001	1.015 (1.013–1.018)	< 0.001
			**CAD**			
Quartile 1	Ref		Ref		Ref	
Quartile 2	1.15 (1.07–1.23)	< 0.001	1.19 (1.11–1.27)	< 0.001	1.13 (1.06–1.21)	< 0.001
Quartile 3	1.11 (1.03–1.19)	0.004	1.20 (1.12–1.29)	< 0.001	1.11 (1.03–1.19)	0.005
Quartile 4	1.14 (1.07–1.22)	< 0.001	1.39 (1.30–1.48)	< 0.001	1.18 (1.11–1.27)	< 0.001
P for trend	< 0.001		< 0.001		< 0.001	
Per unit increase	1.007 (1.004–1.011)	< 0.001	1.021 (1.017–1.025)	< 0.001	1.011 (1.007–1.015)	< 0.001
			**Stroke**			
Quartile 1	Ref		Ref		Ref	
Quartile 2	1.17 (1.03–1.34)	0.019	1.18 (1.04–1.35)	0.013	1.14 (1.0–1.31)	0.051
Quartile 3	1.15 (1.0–1.31)	0.046	1.20 (1.05–1.37)	0.009	1.11 (0.97–1.28)	0.124
Quartile 4	1.21 (1.07–1.37)	0.002	1.41 (1.25–1.61)	< 0.001	1.24 (1.09–1.42)	0.001
P for trend	< 0.001		< 0.001		0.003	
Per unit increase	1.010 (1.003–1.017)	0.007	1.021 (1.013–1.029)	< 0.001	1.012 (1.004–1.020)	0.003
			**T2DM**			
Quartile 1	Ref		Ref		Ref	
Quartile 2	1.16 (1.09–1.25)	< 0.001	1.24 (1.16–1.33)	< 0.001	1.05 (0.98–1.13)	0.203
Quartile 3	1.34 (1.25–1.43)	< 0.001	1.48 (1.38–1.59)	< 0.001	1.22 (1.13–1.30)	< 0.001
Quartile 4	1.71 (1.61–1.82)	< 0.001	2.04 (1.91–2.17)	< 0.001	1.38 (1.29–1.48)	< 0.001
P for trend	< 0.001		< 0.001		< 0.001	
Per unit increase	1.037 (1.033–1.041)	< 0.001	1.050 (1.046–1.053)	< 0.001	1.024 (1.020–1.028)	< 0.001

HR: hazard ratio; CI: confidence interval; INFLA: the aggregated inflammation score; CMDs: cardiometabolic diseases; T2DM: type 2 diabetes mellitus; CAD: coronary artery disease; BMI: body mass index; SBP: systolic blood pressure; DBP: diastolic blood pressure; TC: total cholesterol; TG: triglyceride; LDL-C: low-density lipoprotein cholesterol; HDL-C: high-density lipoprotein cholesterol

Model 1 is a crude model with no variables being adjusted

Model 2 adjusted for age, sex, and race

Model 3 adjusted for sex, race, age, hypertension, Townsend deprivation index, BMI, DBP, SBP, physical activity, TC, HbA1c, HDL, LDL, Diet score, TG, smoking and alcohol status, antihypertensive, lowering lipids

**Fig. 3:**
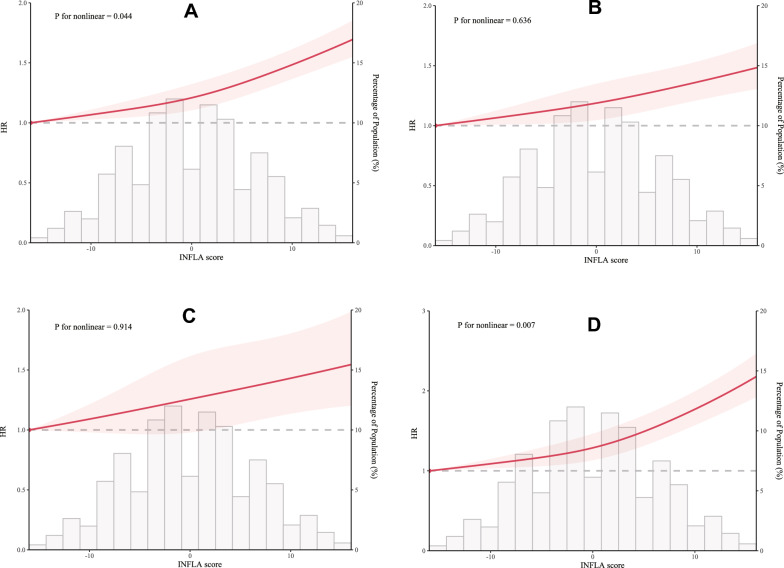
Association of the INFLA-score with CMDs risk using RCS with 3 knots. **A** Cardiometabolic diseases (CMDs). **B** Coronary heart disease (CAD). **C** Stroke. **D** Type 2 diabetes mellitus (T2DM). Hazard ratios were adjusted for the same variables included in model 3. RCS: restricted cubic spline

RCS analysis revealed a positive dose–response relationship between INFLA-score and the risks of CMDs, CAD, stroke and T2DM, with significant non-linear associations for CMDs (P non-linear = 0.044) and T2DM (P non-linear = 0.007), and linear associations between CAD (P non-linear = 0.636) and stroke (P non-linear = 0.914) risk (Fig. 3).

Subsequently, a two-piecewise linear regression model identified inflection points at 8 for CMDs and -2 for T2DM (Table S4). Specifically, when INFLA was < 8, the risk of CMDs increases by 1.3% (HR: 1.013, 95% CI 1.009–1.016) per one unit of INFLA-score and by 3.7% (HR: 1.037, 95% CI 1.022–1.052) per one unit of INFLA-score when INFLA-score is ≥ 8. Moreover, when INFLA-score ≥ − 2, a positive correlation between INFLA-score and T2DM incidence was observed (HR: 1.031, 95% CI 1.025–1.036), while no correlation was observed between them when INFLA-score < − 2 (P = 0.084, Table S2).

**Fig. 4:**
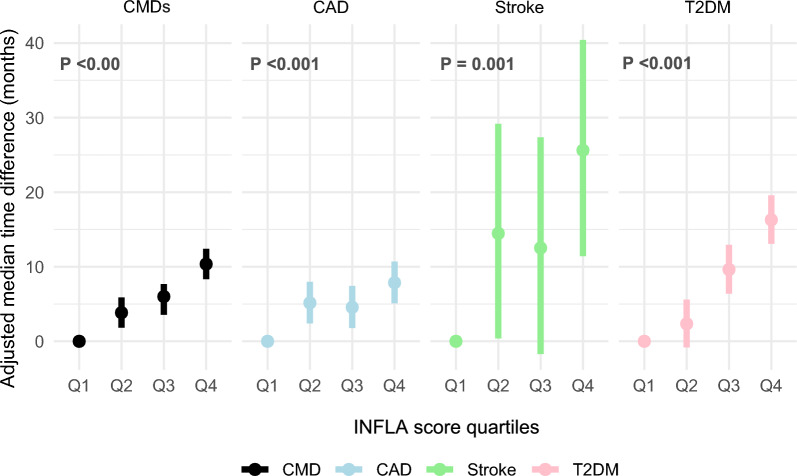
Association of INFLA-score with CMDs risk using AFT. **A** Cardiometabolic diseases (CMDs). **B** Coronary heart disease (CAD). **C** Stroke. **D** Type 2 diabetes mellitus (T2DM). Hazard ratios were adjusted for the same variables included in model 3. AFT: accelerated time-to-failure

Finally, AFT models revealed that higher INFLA-scores were significantly associated with the earlier onset of CMDs, CAD, stroke and T2DM compared to the lowest quartile of INFLA-scores (all P < 0.01; Fig. 4).

### Subgroup analysis and sensitivity analyses

**Fig. 5:**
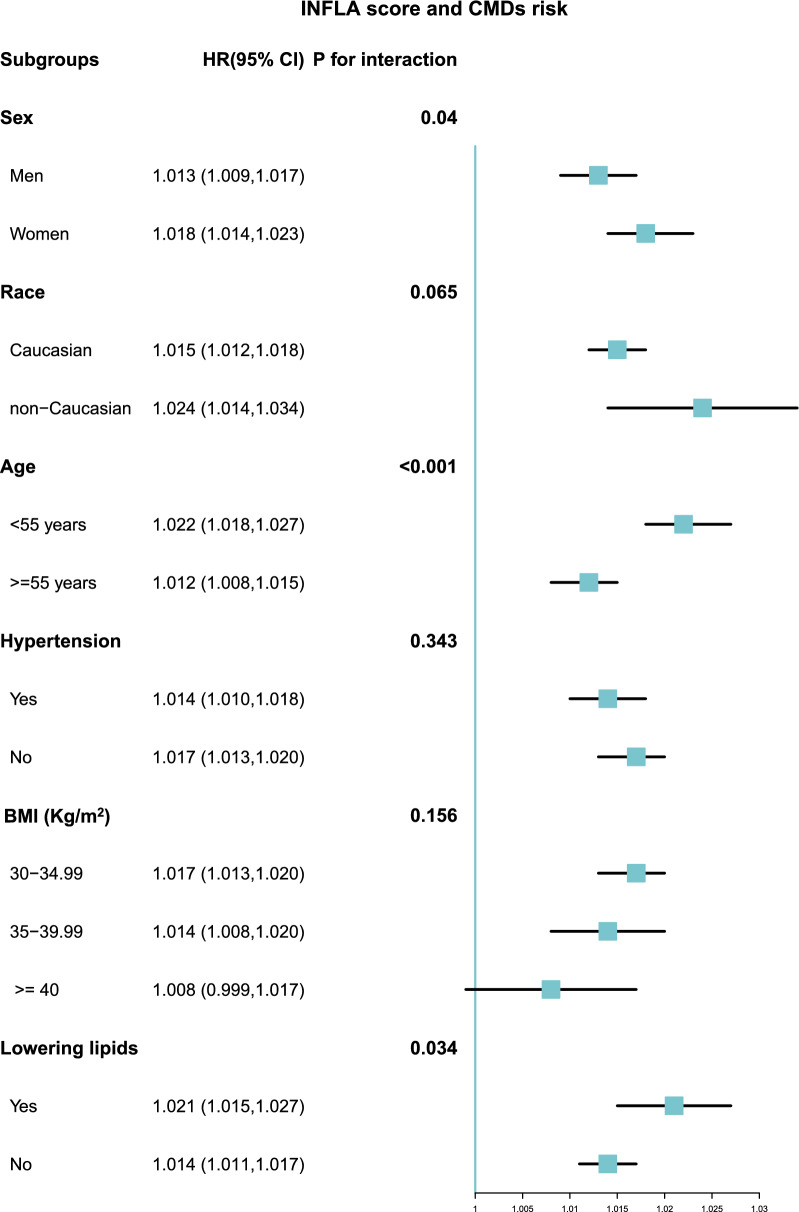
Subgroup and interaction analysis between INFLA-score and CMDs across various subgroups. CMDs: Cardiometabolic diseases;

Stratified analyses were conducted based on sex, ethnicity, age, history of hypertension, BMI and history of lipid-lowering drug use (Fig. 5). The results revealed that an increase in INFLA-score was associated with an increased risk of CMDs, except in participants with a BMI ≥ 40 kg/m^2^. Similarly, significant interactions were observed for sex, age and history of lipid-lowering drug use (p-value for interaction < 0.05).

In the sensitivity analysis, we conducted a reanalysis after including 1774 participants within the first 2 years of follow-up, which yielded results consistent with the main findings (Table S3). Additionally, we performed another analysis using multiple imputations, wherein the pooled results were similar to the main results (Table S4).

## Discussion

To the best of our knowledge, this study is the first large-scale prospective cohort to investigate the relationship between INFLA-score and the risk of CMDs. Our findings demonstrate that individuals with higher INFLA-scores are associated with an elevated incidence of CMDs, encompassing CAD, stroke and T2DM. Notably, this association remained statistically significant even after adjusting for established CMDs risk factors. Furthermore, RCS revealed a dose–response relationship between INFLA-score and CMDs risk, with a notable surge in CMDs risk observed when the INFLA-score ≥ 8. Subgroup analyses further supported a consistent elevation in CMDs risk with increasing INFLA-scores across various demographic populations. Furthermore, sensitivity analyses reinforced the robustness of our primary findings.

hsCRP stands out as a pivotal marker of systemic inflammation and has shown a strong linear correlation with cardiovascular event occurrence [25]. Moreover, studies have demonstrated that following anti-inflammatory interventions, reductions in hsCRP levels coincide with diminished rates of cardiovascular events [26]. Chen et al. indicated that leukocyte count (OR = 1.50; 95% CI = 1.25–1.81) is an independent risk factor for IR after adjusting for confounding variables [27]. Increasing evidence also suggests that both leukocyte count and IR are significant pathological indicators for the occurrence and development of Mets such as T2DM and CADs [28, 29]. The platelet count was found to be significantly higher in populations with Mets compared to those without Mets [30]. Additionally, an elevated baseline platelet count has been reported to significantly contribute to the onset of diabetes [31]. Furthermore, neutrophil-to-lymphocyte ratio (NLR), representing a primary granulocyte component, has been implicated in conditions like CAD and T2DM, highlighting its relevance as an indicator of CMDs [32, 33].

The precise mechanistic link between INFLA-score and CMDs remains elusive. Nonetheless, evidence suggests that obesity drives an increase in hs-CRP levels [34], which in turn hinders insulin receptor activation [35]. This reduction further leads to a decrease in cell sensitivity to insulin, resulting in IR and subsequently contributing to the occurrence of Mets such as T2DM [35]. Moreover, elevated hs-CRP levels stimulate inflammatory responses, fostering endothelial dysfunction and atherosclerosis progression, thereby heightening CVD risk [36]. Notably, anti-inflammatory interventions have demonstrated reductions in hsCRP levels, concomitant with decreased cardiovascular event rates [26]. Furthermore, inflammatory factors such as interleukin-6 and leptin promote leukocyte proliferation and differentiation [37], exacerbating the inflammatory response and leading to vasodilation, nitric oxide suppression and prostacyclin production, thereby reducing the endothelium's anti-thrombotic and anti-atherosclerotic functions [38, 39]. This results in arterial wall damage and plaque formation, subsequently causing thrombosis and leading to cardiovascular events such as myocardial infarction and stroke [38]. Additionally, a high leukocyte count can directly bind to the vascular endothelium, leading to an increase in leukocytes in capillaries [40]. Subsequently, this may cause capillary narrowing and increased vascular pressure, ultimately leading to hypertension and cardiovascular events [40]. Moreover, platelets release various inflammatory mediators such as platelet factor 4 and platelet-activating factor, which can trigger inflammatory responses and promote the release of inflammatory mediators from vascular endothelial cells [41]. Platelets also interact with leukocytes, facilitating their adhesion and migration, thus amplifying the inflammatory response [42, 43]. Platelet activation and aggregation play critical roles in atherosclerotic plaque formation and stabilisation. These processes not only expedite plaque formation but also increase the risk of plaque rupture, triggering CMDs events [44]. Neutrophils and lymphocytes also contribute significantly to inflammatory responses and CMDs pathogenesis. Neutrophils engage in inflammation through various mechanisms such as reactive oxygen species release and extracellular trap formation, fostering interactions with vascular endothelial cells and platelets to promote immune thrombosis and ensuing cardiovascular events [45, 46]. Conversely, lymphocytes possess regulatory functions, with regulatory T cells potentially inhibiting atherosclerosis, while B lymphocyte deficiency can exacerbate atherosclerosis, culminating in CMDs [47–49].

We observed that the risk of T2DM increased more significantly with each unit increase in the INFLA score compared to CAD and stroke. This could be because the INFLA score reflects the level of chronic inflammation in the body, which is closely associated with insulin resistance and impaired β-cell function, thereby increasing the risk of diabetes [50]. hs-CRP, an acute-phase reactive protein, often indicates a systemic inflammatory response when elevated. High white blood cell counts and increased GrL are also associated with chronic inflammation [51]. These inflammatory markers may work together to promote the development of T2DM. In addition, regarding the time to disease onset, the onset of stroke occurred significantly earlier with increasing INFLA score quartiles. This suggests that high levels of chronic inflammation are strongly associated with earlier stroke onset. The occurrence of stroke is closely related to vascular endothelial damage and the formation and rupture of atherosclerotic plaques. High levels of hs-CRP, increased white blood cell counts, and elevated GrL in INFLA scores indicate enhanced inflammation and immune responses within the vasculature. These factors may lead to the accelerated progression of atherosclerosis and the formation of unstable plaques, thereby increasing the risk of stroke [52, 53]. However, while CAD is associated with atherosclerosis, its onset and progression are more significantly influenced by abnormal lipid metabolism, hypertension, and other metabolic syndrome factors. The impact of chronic inflammation on CAD may take longer to manifest.

Subgroup analysis revealed interactions between the INFLA-score and CMDs risk regarding sex, age and history of lipid-lowering drug use. Compared to male participants, the association between INFLA-score and CMDs appeared more pronounced in female participants, suggesting a higher risk of CMDs among women, consistent with previous studies [54]. Additionally, individuals aged < 55 exhibited a stronger association between INFLA-score and CMDs compared to those aged ≥ 55 years, although the underlying mechanisms remain unclear. We speculate that in obese elderly individuals, the immune system may adapt to chronic inflammation levels, while older individuals are more likely to adopt healthier lifestyles and maintain appropriate weight, mitigating metabolic disease risk [55]. Furthermore, individuals with a history of lipid-lowering drug use displayed increased susceptibility to CMDs compared to non-users. Similarly, Sattar et al. reported a slight increase in diabetes risk associated with statin therapy, consistent with our observations [56].

The INFLA-score quantifies inflammation levels, crucial in the onset and progression of CMDs. Utilizing the INFLA-score aids physicians in assessing patients' overall health and CMDs risk. For screening, the INFLA-score serves as an additional indicator to detect underlying inflammatory states. Early detection and intervention can prevent CMDs, improving patients' prognosis. In risk assessment, the INFLA-score complements traditional indicators like blood pressure, blood sugar, and lipid levels, providing a comprehensive risk profile. This approach identifies individuals with chronic inflammation, enabling targeted preventive measures to reduce CMDs incidence and progression. However, incorporating the INFLA-score into routine practice requires further research and clinical validation to confirm its accuracy, reliability, and utility.

By applying the INFLA-score, high-risk individuals, particularly those with obesity, can be identified early, allowing for interventions before severe symptoms manifest and preventing CMDs. The INFLA-score supports personalized prevention strategies: high-score individuals receive aggressive lifestyle interventions and monitoring, while low-score individuals benefit from routine health education. This personalized approach effectively manages obesity and CMDs risk. Our study also raises public awareness about the link between obesity and CMDs, highlighting the role of low-grade inflammation and promoting healthy lifestyles, such as balanced diets, moderate exercise, and avoiding smoking and excessive drinking. The research provides a scientific basis for public health policies, encouraging community health plans, obesity prevention programs, and regular health screenings.

Adjusting anti-inflammatory, lipid-lowering, or glucose-lowering medications based on the INFLA-score effectively controls inflammation and metabolic disturbances. Tailored diet and exercise plans further reduce inflammation and improve metabolic health. Regular INFLA-score monitoring allows dynamic adjustments to treatment plans, ensuring maximal therapeutic effects and timely complication management. Interdisciplinary collaboration involving endocrinologists, cardiologists, nutritionists, and psychologists is essential for comprehensive treatment plans, enhancing overall efficacy and improving the health and quality of life of obese patients. Finally, INFLA-score research may inspire the development of novel therapies targeting specific inflammatory mechanisms to prevent CMDs.

However, several limitations warrant consideration. Firstly, the participants were exclusively from the UK, with the majority being Caucasian, limiting the generalizability of our findings to other regions and ethnicities. Secondly, despite efforts to control for confounding factors influencing CMDs, residual confounding effects may persist. Lastly, the use of baseline INFLA-scores makes it challenging to infer the dynamic impact of INFLA-score fluctuations on CMDs occurrence during follow-up. Besides, in the secondary outcome of this study, stroke could not be analysed by further differentiating subtypes due to limitations of the current data.

## Conclusion

The INFLA-score, serving as a comprehensive inflammation marker, exhibits a robust association with elevated risk and accelerated onset of CMDs among individuals with obesity. By calculating the INFLA-score in these populations, healthcare professionals can gauge CMDs risk levels and pinpoint vulnerable populations. Leveraging the INFLA-score can facilitate the tailoring of personalised prevention and management strategies, thereby mitigating CMDs incidence in patients with obesity.

### Supplementary Information

Supplementary Material 1.

## Data Availability

Data can be accessed from a public and open repository. This study was conducted using the UK Biobank Resource, Application Number: 107335. Interested researchers can apply for access to the UK Biobank data at www.ukbiobank.ac.uk.
