# Supplementary material for: INFLA score: a novel inflammatory marker for assessing cardiometabolic disease risk in obese individuals
Source: Diabetol Metab Syndr. 2024 Jul 9;16:151. doi: 10.1186/s13098-024-01396-8 (PMC11232261; doi:10.1186/s13098-024-01396-8)
Supplement: Supplementary file 1 — Supplementary Material 1. [file 13098_2024_1396_MOESM1_ESM.pdf]

Association between chronic low-grade inflammation and risk of of cardiovascular metabolic diseases: a prospective cohort study in the UK Biobank

Table S1. Baseline characteristics of the overall population. 2
Table S2. Multivariate COX regression analyses of the association between INFLA score and their constituent inflammatory indicators per standard deviation increase and risk of CMDs………..……3
Table S3. Threshold-effect analysis on INFLA-score and the risk of cardiometabolic diseases. 3
Table S4. Multivariate COX regression analyses of INFLA score and CMDs risk *. 4
Table S5. Multivariate COX regression of one score increase in INFLA score and CMDs risk after multiple imputations of five data sets. 5
Figure S1. Histogram of the distribution of Time to CMDs. CMM, cardiometabolic diseases. 6
Figure S2. Normal Q-Q Plot of time to cardiometabolic diseases..7
Appendix 1: Identifying inflection points for threshold effect analysis 8

Table S1. Baseline characteristics of the overall population.

Age	56 (49, 62)
Men (%)	35929 (45.39%)
Caucasian (%)	74208 (94.24%)
Current smoker (%)	7411 (9.41%)
Current drinker (%)	71928 (91.13%)
Hypertension (%)	28931 (36.55%)
Antihypertensive (%)	20381 (25.78%)
Lowering lipids (%)	11846 (14.98%)
TDI	-1.80 (-3.44, 1.08)
BMI	32.59 (31.09,35.12)
DBP	86.50 (80.00, 92.50)
SBP	140.50 (129.50, 152.50)
Physical activity	2364.68 (872, 2388)
TC	5.76 (5.05, 6.52)
HBA1C	36.10 (33.50, 38.30)
HDL	1.30 (1.10, 1.50)
LDL	3.68 (3.13, 4.26)
Diet score	5.00 (4.00, 6.00)
Glucose	5.02 (4.67, 5.31)
TG	1.85 (1.32, 2.58)
**Inflammation related biomarkers**	
C-reactive protein (mg/L)	2.53 (1.39,4.71)
Lymphocyte count ($10^{\wedge 9}$ cells/L)	2.00 (1.65,2.42)
Neutrophil count ($10^{\wedge 9}$ cells/L)	4.25 (3.48, 5.20)
Eosinophil count ($10^{\wedge 9}$ cells/L)	0.16 (0.10, 0.23)
Basophil count ($10^{\wedge 9}$ cells/L)	0.02 (0.00, 0.05)
GrL	2.22 (1.75, 2.82)
Leucocyte count ($10^{\wedge 9}$ cells/L)	7.06 (6.00, 8.26)
Platelet count ($10^{\wedge 9}$ cells/L)	253.10 (217.50, 293.40)
**INFLA-score Components**	
GrL score	0.00 (-2.00, 2.00)
WHITE_SCORE continuous	0.00 (-2.00, 2.00)
PLATELET_SCORE continuous	0.00 (-2.00, 2.00)
C-reactive protein score	0.00 (-2.00, 2.00)

INFLA, the aggregated inflammation score. TDI, Townsend Deprivation Index. BMI, body mass index. SBP, systolic blood pressure. DBP, diastolic blood pressure. TC, total cholesterol. TG, triglyceride. LDL-C, low-density lipoprotein cholesterol. HDL-C, high-density lipoprotein cholesterol. MET, metabolic equivalent task. GrL, granulocyte to lymphocyte ratio.

Table S2. Multivariate COX regression analyses of the association between INFLA score and their constituent inflammatory indicators per standard deviation increase and risk of CMDs.

	HR (95% CI)	P value
**INFLA score**	1.099 (1.080-1.118)	<0.001
hs-CRP	1.073 (1.059-1.088)	<0.001
leukocyte count	1.096 (1.079-1.113)	<0.001
platelet	0.999 (0.982-1.017)	0.945
granulocyte/lymphocyte ratio	1.027 (1.016-1.039)	<0.001

HR has been fully adjusted for the following variables: sex, race, age, hypertension, Townsend deprivation index, BMI, DBP, SBP, physical activity, TC, HbA1c, HDL, LDL, Diet score, TG, smoking and alcohol status, antihypertensive, lowering lipids.

Table S3. Threshold-effect analysis on INFLA-score and the risk of cardiometabolic diseases.

Inflection-point of INFLA-score	HR	95% CI	P-value	P for nonlinear
**CMD**				0.044
＜8	1.013	1.009-1.016	＜0.001	
≥8	1.037	1.022-1.052	＜0.001	
**T2DM**				0.007
＜-2	1.009	0.999-1.019	0.084	
≥-2	1.031	1.025-1.036	＜0.001	

HR, hazard ratio. CI, confidence interval.

HR has been fully adjusted for the following variables: sex, race, age, hypertension, Townsend deprivation index, BMI, DBP, SBP, physical activity, TC, HbA1c, HDL, LDL, Diet score, TG, smoking and alcohol status, antihypertensive, lowering lipids.

Table S4. Multivariate COX regression analyses of INFLA score and CMDs risk *.

	HR (95% CI)	P value
**INFLA score**	**CMDs**	
Quartile 1	Ref	
Quartile 2	1.10 (1.05-1.15)	<0.001
Quartile 3	1.13 (1.08-1.18)	<0.001
Quartile 4	1.26 (1.20-1.31)	<0.001
P for trend	<0.001	
Per unit increase	1.016 (1.013-1.018)	<0.001
	**CAD**	
Quartile 1	Ref	
Quartile 2	1.13 (1.06-1.21)	<0.001
Quartile 3	1.11 (1.04-1.18)	0.002
Quartile 4	1.20 (1.13-1.28)	<0.001
P for trend	<0.001	
Per unit increase	1.011 (1.008-1.015)	<0.001
	**Stroke**	
Quartile 1	Ref	
Quartile 2	1.15 (1.01-1.30)	0.037
Quartile 3	1.10 (0.97-1.26)	0.137
Quartile 4	1.28 (1.13-1.45)	<0.001
P for trend	<0.001	
Per unit increase	1.014 (1.006-1.021)	<0.001
	**T2DM**	
Quartile 1	Ref	
Quartile 2	1.04 (0.97-1.12)	0.217
Quartile 3	1.21 (1.13-1.30)	<0.001
Quartile 4	1.38 (1.29-1.47)	<0.001
P for trend	<0.001	
Per unit increase	1.024 (1.020-1.028)	<0.001

*The analysis included 1774 participants who were excluded within two years.

INFLA, the aggregated inflammation score. CAD, Cardiometabolic Diseases. T2DM, Type 2 diabetes mellitus. CAD, Coronary artery disease.

Table S5. Multivariate Cox regression of one score increase in INFLA score and CMDs risk after multiple imputations of five data sets.

	Data 1	Data 2	Data 3	Data 4	Data 5	Pooled results	
	HR (95% CI)	HR (95% CI)	HR (95% CI)	HR (95% CI)	HR (95% CI)	HR (95% CI)	P value
				CMD			
Quartile 1	Ref	Ref	Ref	Ref	Ref	Ref	
Quartile 2	1.09 (1.04-1.15)	1.09 (1.04-1.14)	1.09 (1.04-1.15)	1.09 (1.04-1.14)	1.09 (1.04-1.14)	1.09 (1.04-1.14)	<0.001
Quartile 3	1.13 (1.07-1.18)	1.12 (1.07-1.18)	1.12 (1.07-1.18)	1.12 (1.07-1.18)	1.12 (1.07-1.18)	1.12 (1.07-1.18)	<0.001
Quartile 4	1.24 (1.19-1.30)	1.24 (1.18-1.30)	1.25 (1.19-1.31)	1.24 (1.19-1.30)	1.24 (1.19-1.30)	1.24 (1.19-1.30)	<0.001
P for trend	<0.001	<0.001	<0.001	<0.001	<0.001	<0.001	
Per unit increase	1.015 (1.012-1.018)	1.015 (1.012-1.018)	1.015 (1.012-1.018)	1.015 (1.012-1.018)	1.015 (1.012-1.018)	1.015 (1.012-1.018)	<0.001
				CAD			
Quartile 1	Ref	Ref	Ref	Ref	Ref	Ref	
Quartile 2	1.13 (1.06-1.21)	1.13 (1.06-1.21)	1.13 (1.06-1.21)	1.13 (1.06-1.21)	1.13 (1.06-1.21)	1.13 (1.06-1.21)	<0.001
Quartile 3	1.11 (1.03-1.18)	1.10 (1.03-1.18)	1.10 (1.03-1.18)	1.10 (1.03-1.18)	1.11 (1.03-1.18)	1.10 (1.03-1.18)	0.005
Quartile 4	1.19 (1.11-1.27)	1.19 (1.11-1.27)	1.19 (1.11-1.27)	1.19 (1.11-1.27)	1.19 (1.11-1.27)	1.19 (1.11-1.27)	<0.001
P for trend	<0.001	<0.001	<0.001	<0.001	<0.001	<0.001	
Per unit increase	1.011 (1.007-1.015)	1.011 (1.007-1.015)	1.011 (1.007-1.015)	1.011 (1.007-1.015)	1.011 (1.007-1.015)	1.011 (1.007-1.015)	<0.001
				Stroke			
Quartile 1	Ref	Ref	Ref	Ref	Ref	Ref	
Quartile 2	1.13 (0.99-1.30)	1.13 (0.99-1.29)	1.13 (0.99-1.29)	1.13 (0.99-1.29)	1.13 (0.99-1.30)	1.13 (0.99-1.29)	0.072
Quartile 3	1.12 (0.97-1.28)	1.11 (0.97-1.28)	1.12 (0.97-1.28)	1.11 (0.97-1.27)	1.11 (0.97-1.28)	1.11 (0.97-1.28)	0.137
Quartile 4	1.24 (1.09-1.41)	1.24 (1.09-1.41)	1.24 (1.09-1.41)	1.23 (1.08-1.41)	1.24 (1.09-1.41)	1.24 (1.09-1.41)	<0.001
P for trend	0.003	0.003	0.003	0.003	0.003	<0.001	
Per unit increase	1.012 (1.005-1.020)	1.012 (1.004-1.020)	1.012 (1.005-1.020)	1.012 (1.004-1.020)	1.012 (1.004-1.020)	1.012 (1.004-1.020)	<0.001
				T2DM			
Quartile 1	Ref	Ref	Ref	Ref	Ref	Ref	
Quartile 2	1.05 (0.98-1.13)	1.05 (0.97-1.12)	1.04 (0.97-1.12)	1.04 (0.96-1.11)	1.04 (0.97-1.11)	1.04 (0.97-1.12)	0.255
Quartile 3	1.21 (1.12-1.29)	1.20 (1.12-1.29)	1.20 (1.12-1.29)	1.20 (1.12-1.29)	1.20 (1.12-1.29)	1.20 (1.12-1.29)	<0.001
Quartile 4	1.36 (1.27-1.45)	1.36 (1.28-1.45)	1.37 (1.28-1.46)	1.37 (1.28-1.46)	1.37 (1.28-1.46)	1.37 (1.28-1.46)	<0.001
P for trend	<0.001	<0.001	<0.001	<0.001	<0.001	<0.001	
Per unit increase	1.023 (1.019-1.027)	1.023 (1.019-1.027)	1.024 (1.020-1.028)	1.024 (1.020-1.028)	1.024 (1.020-1.028)	1.024 (1.020-1.028)	<0.001

INFLA, the aggregated inflammation score. CAD, Cardiometabolic Diseases. T2DM, Type 2 diabetes mellitus. CAD, Coronary artery disease.

Figure S1. Histogram of the distribution of Time to CMDs. CMM, cardiometabolic diseases.

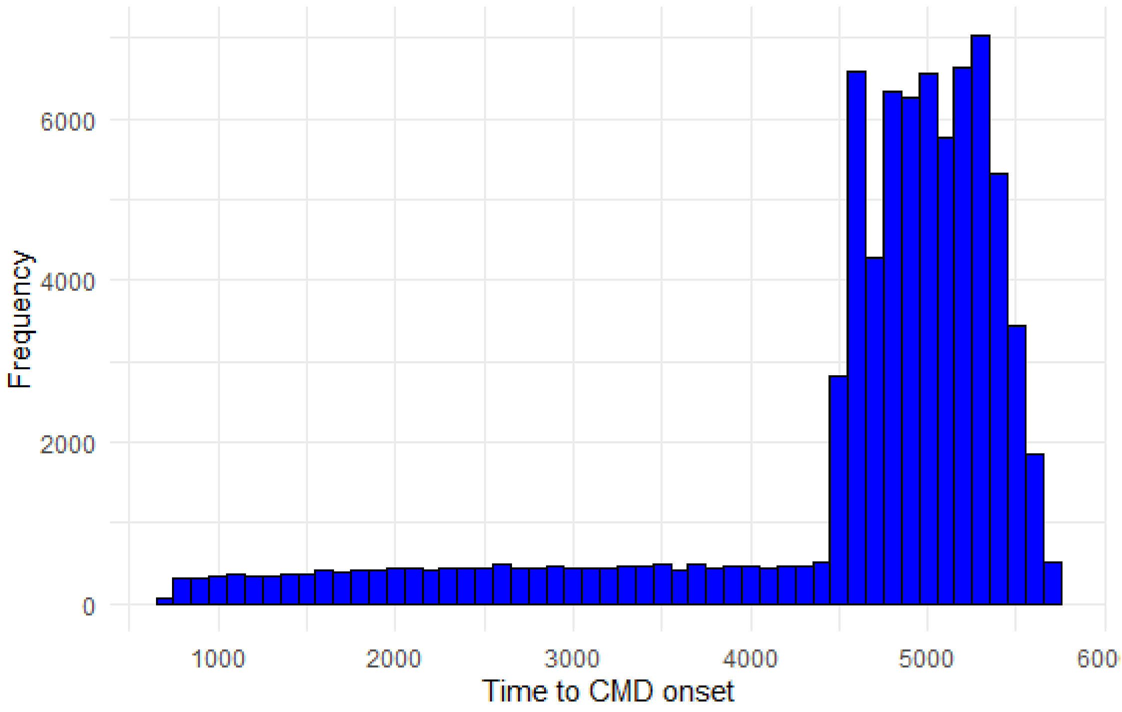

Figure S2. Normal Q-Q Plot of time to cardiometabolic diseases.

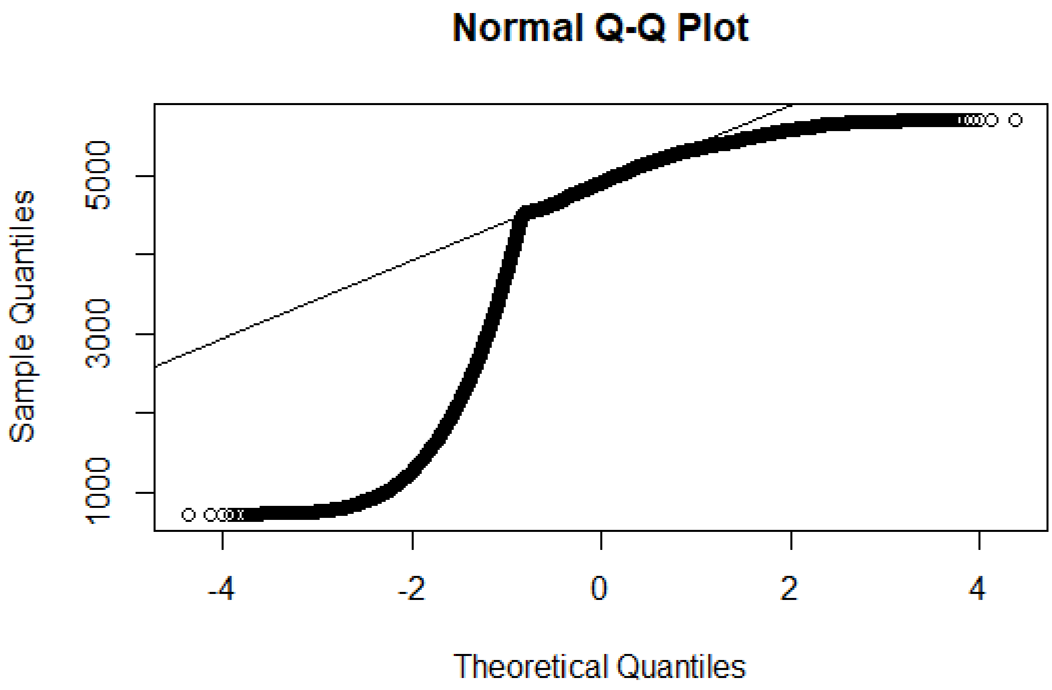

Appendix 1: Identifying inflection points for threshold effect analysis

Our approach begins with the use of restricted cubic splines to explore the non-linear behavior of the independent variable, with the aim of identifying possible divisions into distinct segments. We then apply segmented regression - also known as piecewise regression. This technique involves fitting linear segments to the identified segments. To assess the presence of a threshold effect, we perform a log-likelihood ratio test comparing the conventional one-line model with the segmented model. The process of identifying the inflection point that connects these segments, based on the principle of maximum likelihood, is carried out in two main steps.

In the first stage, we focus on narrowing the range of the potential inflection point to a 10-percentile range of the independent variable. By evaluating 19 segmented regression models at percentile intervals ranging from 5% to 95%, we identify the percentile that maximizes likelihood. We then refine the location of the inflection point to within +/- 4% of this optimal percentile, referred to as Kmin and Kmax.

The next stage focuses on accurately locating the inflection point between Kmin and Kmax using a recursive technique. This involves running three preliminary models with potential inflection points at the 25th (Q1), 50th (Q2) and 75th (Q3) percentiles within the defined range. The model with the highest probability among these sets the new bounds for Kmin and Kmax, adjusting them to within +/- 25% of the identified quartile. This recursive narrowing process is repeated until a precise value for the independent variable is identified. Using this value as the inflection point optimizes the likelihood of the segmented regression model.
